# Analysis of BRAF^V600E^ mutation and DNA methylation improves the diagnostics of thyroid fine needle aspiration biopsies

**DOI:** 10.1186/1746-1596-9-45

**Published:** 2014-03-03

**Authors:** Bingfei Zhang, Shu Liu, Zhaoxia Zhang, Jing Wei, Yiping Qu, Kexia Wu, Qi Yang, Peng Hou, Bingyin Shi

**Affiliations:** 1 Department of Endocrinology, The First Affiliated Hospital of Xi’an Jiaotong University School of Medicine, Xi’an 710061, P. R. China

**Keywords:** *BRAF*^*V600E*^ mutation, DNA methylation, Fine needle aspiration biopsy (FNAB), Thyroid nodules, Papillary thyroid cancer (PTC)

## Abstract

**Background:**

Thyroid nodules with indeterminate cytological features on fine needle aspiration biopsy specimens (FNABs) have a ~20% risk of thyroid cancer. *BRAF*^*V600E*^ mutation and DNA methylation are useful markers to distinguish malignant thyroid neoplasm from benign. The aim of this study was to determine whether combined detection of *BRAF*^*V600E*^ mutation and methylation markers on FNABs could improve the diagnostic accuracy of thyroid cancer.

**Methods:**

Using pyrosequencing and quantitative methylation-specific PCR (Q-MSP) methods, FNABs from 79 and 38 patients with thyroid nodules in training and test groups, respectively, were analyzed for *BRAF*^*V600E*^ mutation and gene methylation.

**Results:**

*BRAF*^*V600E*^ mutation was found in 30/42 (71.4%) and 14/20 (70%) FNABs in training and test groups, respectively. All *BRAF*^*V600E*^-positive samples were histologically diagnosed as papillary thyroid cancer (PTC) after thyroidectomy. As expected, *BRAF* mutation was not found in all benign nodules. Moreover, we demonstrated that the five genes, including *CALCA*, *DAPK1*, *TIMP3*, *RAR-beta* and *RASSF1A*, were aberrantly methylated in FNABs. Of them, methylation level of *DAPK1* in PTCs was significantly higher than that in benign samples (*P* <0.0001). Conversely, methylation level of *RASSF1A* in PTCs was significantly lower than that in benign samples (*P* =0.003). Notably, compared with *BRAF* mutation testing alone, combined detection of *BRAF* mutation and methylation markers increased the diagnostic sensitivity and accuracy of PTC with excellent specificity.

**Conclusion:**

Our data have demonstrated that combine analysis of *BRAF* mutation and DNA methylation markers on FNABs may be a useful strategy to facilitate the diagnosis of malignant thyroid neoplasm, particularly PTC.

**Virtual slides:**

The virtual slide(s) for this article can be found here: http://www.diagnosticpathology.diagnomx.eu/vs/6080878071149177.

## Background

Thyroid cancer is the most common endocrine malignancy. The current rise in the incidence of thyroid cancer is mainly from an increased incidence in papillary thyroid cancer (PTC) [1]. The initial presentation of thyroid cancer is usually a thyroid nodule, which is palpable in approximately 5% of normal adults [1] and visualized by sonography in one third or more of normal adults [2,3]. Given that the prevalence of malignancy in solitary thyroid nodules is only ~5% in adults [4,5], the preoperative differentiation of benign from malignant thyroid nodules is imperative. Fine needle aspiration biopsy (FNAB) is the most reliable nonsurgical test for the diagnosis of thyroid cancer, with high sensitivity and specificity [6,7]. FNAB represents the “gold standard”, and is widely used in the evaluation of thyroid nodules. However, ~20% of FNABs are diagnosed as indeterminate cytological findings that cannot distinguish cancerous from benign neoplasms [8,9].

In addition to routine cytological examination, a number of molecular markers play an important role in the diagnosis of thyroid nodules, such as *BRAF* and *Ras* mutations, and *RET/PTC* rearrangements [10]. Of them, a prominent oncogenic genetic event in PTC is *BRAF*^*V600E*^ mutation. This mutation occurs in the majority of cases and results in the constitutively activated BRAF kinase [11], which plays a fundamental role in thyroid tumorigenesis through driving Ras/Raf/MEK/ERK (MAPK) signaling pathway [10,11]. Notably, *BRAF* mutation has consistently been reported to be specific for PTC, whereas no benign thyroid neoplasms or normal thyroid tissues have been found to harbor this mutation. Thus, *BRAF* mutation has the potential to be a specific molecular marker with relatively high sensitivity for PTC diagnosis. In recent years, accumulated evidences have demonstrated that *BRAF* mutation detection on FNAB specimens (FNABs) enhances the diagnostic value of cytology [12-14].

Epigenetic alterations, such as promoter hypermethylation, are one of the most common molecular events in human cancers, along with genetic alterations, ultimately leading to carcinogenesis, including thyroid cancer [10,15]. Promoter hypermethylation is an important hallmark of cancer cells and is responsible for transcriptional silencing of tumor suppressor genes during tumorigenesis [16,17]. Aberrant DNA methylation usually occurs somatically in cancer cells and can be released into blood circulation, it may thus serve as a specific diagnostic blood test for cancer, including thyroid cancer [18]. Hence it is not difficult to follow that detection of methylated DNA on FNABs may potentially be a useful diagnostic marker for thyroid cancer.

In this study, we detected *BRAF*^*V600E*^ mutation and promoter methylation of a panel of potential tumor suppressor genes on FNABs using pyrosequnecing and quantitative methylation-specific PCR (Q-MSP) approaches, and determined the diagnostic value of combined analysis of these two molecular events on FNABs in the evaluation of thyroid nodules.

## Methods

### Patients and FNAB procedures

With the institutional review board approval and patient consent, we recruited 117 study patients whom we were able to obtain preoperative thyroid FNABs for *BRAF* mutation and DNA methylation analysis at the First Affiliated Hospital of Xi’an Jiaotong University School of Medicine between August 2011 and December 2012. These patients were chosen for this study because their thyroid nodule was easily palpable for fine needle aspiration and they were considered for thyroid surgery. To find appropriate cut-off values that can distinguish between malignant and benign nodules, 79 patients were classified into the training group, including 42 cases with PTC and 37 cases with benign nodules. As an independent test group, FNABs were collected from 38 patients with thyroid nodule. Clinicopathological data of the patients were summarized in Table 1.

**Table 1:** Clinicopathological characteristics of the patients with thyroid nodule

**Characteristics**	** No. of patients in training group (%)**		** No. of patients in test group (%)**	
	**PTC (n =42)**	**Benign (n =37)**	**PTC (n =20)**	**Benign (n =18)**
Pathological subtypes				
PTC	42 (100)	--	20 (100)	--
Nodular goiter	--	26 (70.27)	--	14 (77.78)
Thyroid adenoma	--	11 (29.73)	--	4 (22.23)
Gender				
Male	12 (28.57)	9 (24.32)	3 (15.00)	2 (11.11)
Female	30 (71.43)	28 (75.68)	17 (85.00)	16 (88.89)
Age, years				
Mean	45.16	40.31	41.33	40.20
SD	13.17	13.01	12.79	10.45
Tumor/nodule size (cm^3^)				
≤ 3	32 (76.19)	18 (48.65)	14 (70.00)	7 (38.89)
3-5	9 (21.43)	14 (37.84)	6 (30.00)	9 (50.00)
≥5	1 (2.38)	5 (13.51)	0	2 (11.11)
Tumor invasion				
Yes	14 (33.33)	--	14 (70.00)	--
No	28 (66.67)	--	6 (30.00)	--
Lymph node metastasis				
Yes	22 (52.38)	--	15 (75.00)	--
No	20 (47.62)	--	5 (25.00)	--

For the fine needle aspiration procedure, 2 or 3 passes with 21-gauge needles were typically made to harvest material for cytological and molecular analysis. Specimens were collected in 500 μl normal saline solution in a 1.5 ml EP tube. After centrifugation, the pellet was resuspended and washed twice with normal saline solution, and final pellet was used for DNA extraction.

### DNA extraction

Genomic DNA was isolated using a protocol described previously [19]. Briefly, the cells were incubated with 1% sodium dodecyl sulfate (SDS) and 0.5 mg/ml proteinase K at 48°C for 24–48 h. DNA was then isolated by using standard phenol/chloroform protocol, and was dissolved in distilled water and stored at −80°C until use.

### Detection of *BRAF*^*V600E*^ mutation by pyrosequencing assay

A 228-bp region of *BRAF* exon 15 spanning the hotspot mutation site at codon 600 was amplified by PCR using the forward and reverse primers 5′-biotin-CTT CAT AAT GCT TGC TCT GAT AGG-3′ and 5′-GGC CAA AAA TTT AAT CAG TGG AA-3′, respectively. The reaction mixture contained, in a final volume of 25 μl, ~60 ng genomic DNA, 1× PCR buffer, 1.5 mM MgCl_2 ,_ 0.2 mM of each deoxynucleotide triphosphate (dATP, dCTP, dGTP, and dTTP), 0.2 μM each primer (forward and reverse), and 0.6 U platinum DNA *Taq* polymerase (Invitrogen Life Technologies, Inc., MD). The PCR was performed with an initial denaturation at 95°C for 5 min, followed by 35 cycles of 95°C denaturation for 30 s, 58°C annealing for 30 s and 72°C elongation for 30 s. Quality of PCR products was determined by gel electrophoresis. The PCR product was immobilized onto streptavidin-coated sepharose beads (GE Healthcare Bio-Sciences, PA) according to the instructions of the manufacturer. The bead/DNA complex was washed and the supernatant was discarded. Sequencing primer 5′-CCACTCCATCGAGATT-3′ was added and annealed to the captured strand. Pyrosequencing assay was then performed on a PyroMark Q24 system using PyroMark Gold Q24 reagent (Qiagen).

### Sodium bisulfite treatment and quantitative methylation-specific PCR (Q-MSP)

Genomic DNA was subjected to bisulfite treatment as previously described [20]. Briefly, ~2 μg of DNA was denatured by incubation with 0.3 M NaOH at 50°C for 20 min. The denatured DNA was diluted in 500 μl of freshly prepared solution of 10 mM hydroquinone and 3 M sodium bisulfite, and incubated at 80°C for 3 h. The mixture was then purified through a Wizard DNA Clean-Up System (Promega Corp., Madison, WI) according to the instructions of the manufacturer, followed by ethanol precipitation, dry, and resuspension in 100 μl of deionized H_2_O.

Q-MSP assay was performed as described previously [20]. Briefly, quantitative PCR was carried out in a final reaction mixture of 20 μl containing ~3 μl bisulfite-treated DNA, 600 nM each primer, 200 nM TaqMan probe, 5.5 mM MgCl2, 0.6 U platinum Taq polymerase, and 200 μM each of deoxyguanosine triphosphate. The specific primers and TaqMan probes for the target genes and the internal reference gene *β-actin* were presented in Table 2. After an initial denaturation step at 95°C for 2 min, 40 cylces of 15 sec at 95°C and 60 sec at 60°C for annealing and extension were run on a CFX96 Thermal Cycler Dice™ real-time PCR system (Bio-Rad Laboratories, Inc., CA). Normal leukocyte DNA was methylated *in vitro* with *Sss I* methylase (New England Biolabs, Beverly, MA) to generate completely methylated DNA as a positive control. Serial dilutions of completely methylated DNA were used to construct the standard curve. The relative methylation level of each sample was calculated using the method described previously [21]. Each sample was run in triplicate.

**Table 2:** Q-MSP primer and TaqMan probe sequences used in this study

**Genes**	**Forward primer sequences (5′ → 3′)**	**Probe sequence**	**Reverse primer sequence (5′ → 3′)**
*CALCA*	GTTTTGGAAGTATGAGGGTGACG	6FAM-ATTCCGCCAATACACAACAACCAATAAACG-TAMRA	TTCCCGCCGCTATAAATCG
*DAPK1*	GGATAGTCGGATCGAGTTAACGTC	6FAM-TTCGGTAATTCGTAGCGGTAGGGTTTGG-TAMRA	CCCTCCCAAACGCCGA
*TIMP3*	GCGTCGGAGGTTAAGGTTGTT	6FAM-AACTCGCTCGCCCGCCGAA-TAMRA	CTCTCCAAAATTACCGTACGCG
*RAR-beta*	GGGATTAGAATTTTTTATGCGAGTTGT	6FAM-TGTCGAGAACGCGAGCGATTCG-TAMRA	TACCCCGACGATACCCAAAC
*RASSF1A*	GCGTTGAAGTCGGGGTTC	6FAM-ACAAACGCGAACCGAACGAAACCA-TAMRA	CCCGTACTTCGCTAACTTTAAACG
*β-actin*	TGGTGATGGAGGAGGTTTAGTAAGT	6FAM-ACCACCACCCAACACACAATAACAAACACA-TAMR	AAACCAATAAAACCTACTCCTCCCTTAA

### Statistical analysis

Non-parametric Wilcoxon’s Rank Sum test was used for comparison of the differences in methylation levels between malignant and benign nodules because of the significantly skewed distribution of the Q-MSP ratios. The association of gene methylation with *BRAF* mutation was determined using the Spearman’s correlation coefficient. Diagnostic threshold analyses were performed using the receiver operating characteristic (ROC) curves and the areas under the curve (AUC). All statistical analyses were performed using the SPSS statistical package (11.5, Chicago, IL, USA). *P* values <0.05 were considered significant.

## Results

### Detection of *BRAF*^*V600E*^ mutation on FNAB specimens

We used pyrosequencing assay to test *BRAF*^*V600E*^ mutation in a cohort of FNABs from 107 patients with thyroid nodules, which consisted of training and test groups. The former included 42 PTCs and 37 benign nodules. In these PTCs, 30 of 42 (71.43%) cases were positive for *BRAF* mutation. As expected, all benign nodules were negative for *BRAF* mutation. The diagnostic sensitivity of *BRAF* mutation was calculated as the number of PTCs with *BRAF*^*V600E*^-positive divided by the total number of PTCs. The specificity was calculated as the number of benign cases with *BRAF*^*V600E*^-negative divided by the total number of benign cases. Positive predictive value (PPV) was defined as the number of PTCs with *BRAF*^*V600E*^-positive divided by the total number of *BRAF*^*V600E*^-positive cases. Negative predictive value (NPV) was defined as the number of benign cases with *BRAF*^*V600E*^-negative divided by the total number of *BRAF*^*V600E*^-negative cases. As shown in Table 3, the sensitivity, specificity, PPV, NPV, and diagnostic accuracy of *BRAF* mutation detection on FNABs was 71.43%, 100%, 100%, 75.51%, and 71.43%, respectively.

**Table 3:** **Diagnostic values of *BRAF *** **mutation and various DNA methylation markers testing on FNAB specimens for PTC detection [percent (PTCs with *BRAF *** **mutation or methylation-positive/total PTCs, sensitivity; benign nodules with *BRAF *** **mutation or methylation-negative/total benign nodules, specificity; PTCs with *BRAF *** **mutation or methylation-positive/total positive cases, PPV; benign nodules with *BRAF *** **mutation or methylation-negative/total negative nodules, NPV)]**

**Genes**	**Cut-off values**	**Sensitivity (%)**	**Specificity (%)**	**PPV (%)**	**NPV (%)**	**Accuracy (%)**
*BRAF*^*V600E*^	N/A	71.43 (30/42)	100 (37/37)	100 (30/30)	75.51 (37/49)	71.43
*CALCA*	2.405	33.33 (14/42)	81.08 (30/37)	66.67 (14/21)	51.72 (30/58)	14.41
*DAPK1*	0.003	66.67 (28/42)	83.78 (31/37)	82.35 (28/34)	68.89 (31/45)	50.45
*TIMP3*	1.038	30.95 (13/42)	75.68 (28/37)	59.09 (13/22)	49.12 (28/ 57)	6.63
*RAR-beta*	1.606	30.95 (13/42)	75.68 (28/37)	59.09 (13/22)	49.12 (28/57)	6.63
*RASSF1A*	5.311	61.90 (26/42)	78.38 (29/37)	76.47 (26/34)	62.22 (28/45)	40.28
All five genes	N/A	88.10 (37/42)	75.68 (28/37)	80.43 (37/46)	84.85 (28/33)	63.69

*PPV*, Positive predictive value; *NPV*, Negative predictive value.

### Quantitative analysis of DNA methylation on FNAB specimens

**Figure 1:**
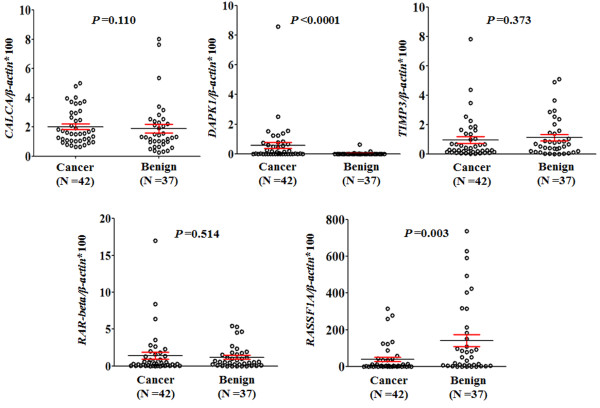
**The overall methylation levels of the five genes in FNAB DNA samples from the patients with thyroid nodules.** Q-MSP assay was performed as described in Methods. The relative methylation level (on *Y* axis) is represented by ratios of candidate gene/*β-actin* (×100). Horizontal lines indicate a 95% confidence interval for the sample mean. *P* values <0.05 were considered significant.

**Figure 2:**
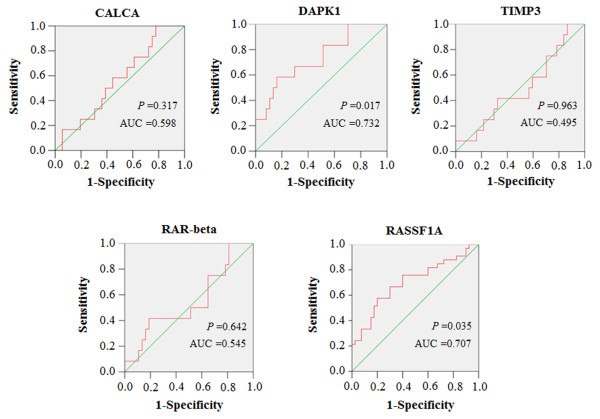
**Receiver operating characteristic (ROC) curves for these five genes.** All malignant and benign thyroid nodules for which there was complete DNA methylation data were used for the analysis. The ROC curves plot sensitivity and 1-specificity. Areas under the curve (AUC) were shown in the graph.

We performed Q-MSP assay on the five genes, *CALCA*, *RAR-beta*, *DAPK1*, *TIMP3,* and *RASSF1A* on FNABs. The differences in methylation levels of these markers between PTCs and benign nodules were illustrated as scatter plots in Figure 1. Among the five genes examined, statistical significances were found in *DAPK1* and *RASSF1A* genes. Methylation level of *DAPK1* in PTCs was significantly higher than that in benign samples (*P* <0.0001). In contrast, *RASSF1A* gene showed lower methylation level in PTCs than that in benign samples (*P* =0.003) (Figure 1). Given that all cases with *BRAF*^*V600E*^-positive were histologically diagnosed as malignant thyroid neoplasm after thyroidectomy, in order to further increase diagnostic sensitivity, the cut-off values for each gene were calculated using ROC curves to distinguish the cases with *BRAF*^*V600E*^-negative from benign nodules (Figure 2). Although AUCs for *DAPK1* (0.732) and *RASSF1A* (0.707) genes were better than other genes, the diagnostic accuracy of these two genes was still low (Table 3), suggesting that these DNA methylation markers play a limited role in the diagnosis of thyroid cancer.

### Correction between *BRAF*^*V600E*^ mutation and DNA methylation

**Figure 3:**
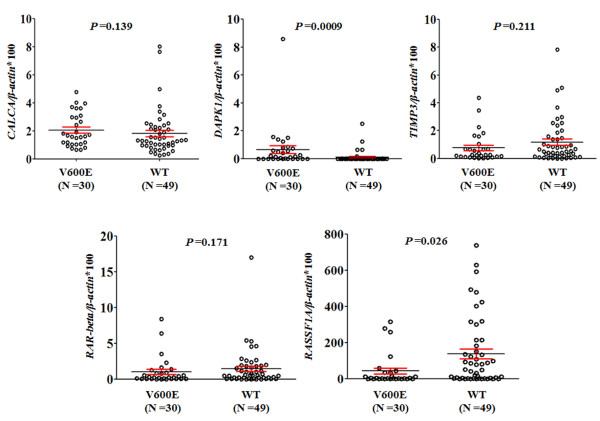
**Association of *BRAF *mutation with methylation levels of these five genes in thyroid tumors.** Shown were scatterplots of methylation ratios in *BRAF* mutation positive (V600E) and negative (WT) neoplastic tumors (including benign and carcinomas). Horizontal lines represent 95% confidence interval. *P* values <0.05 were considered significant.

Given that several previous studies have shown an association of *BRAF*^*V600E*^ mutation with gene methylation in thyroid cancer [21-24], in this study, we also investigated the relationship of DNA methylation levels with this mutation found in these thyroid tumors. In our cohort of 79 thyroid neoplasms, 20 of 40 PTCs and none of benign nodules harbored *BRAF* mutation. Of these five methylation markers investigated, *BRAF* mutation was significantly associated with *DAPK1* methylation (*P* =0.0009), as well as with an absence of *RASSF1A* methylation (*P* =0.026) by Wilcoxon test analysis (Figure 3). The Spearman’s rank correlation analysis confirmed that both of these associations were significant (*P* <0.05).

### Combined detection of *BRAF*^*V600E*^ mutation and methylation markers for improving the diagnostic accuracy of thyroid cancer

We next assessed the usefulness of combined detection of *BRAF* mutation and methylation markers on FNABs in the diagnosis of malignant thyroid neoplasm. We set up appropriate cut-off values to distinguish malignant from benign nodules in the training group, with more focus on diagnostic accuracy for each combination. As shown in Table 4, the specificity of each combination was all 100%. The diagnostic sensitivity, ranging from 71.43 to 78.57%, was obtained for combining *BRAF* mutation with each individual methylation marker. As compared with *BRAF* mutation testing alone (71.43%), the sensitivity and accuracy of combined analysis of each individual gene methylation and *BRAF* mutation were not significantly improved (Table 4). However, with these cut-off values of methylation, the overall diagnostic sensitivity and accuracy of the five genes for thyroid cancer, when at least one of the five genes were all positive, rose to 85.71%, with PPV and NPV of 100 and 86.05%, respectively. Subsequently, we performed *BRAF* mutation detection and Q-MSP assay on FNABs from 38 patients with thyroid nodules (defined as test group), with a blinded histologic diagnosis. Of them, 20 samples were histologically diagnosed as PTC after thyroidectomy. Other samples were diagnosed as benign nodules. Also shown in Table 4, in these PTCs, 14 of 20 (70%) cases were positive for *BRAF*^*V600E*^ mutation. Similarly, all benign nodules were negative for *BRAF*^*V600E*^ mutation. Using the same cut-off values of methylation as for training group, the excellent diagnostic specificity, ranging from 94.44 to 100%, was found for each combination. As compared with *BRAF* mutation testing alone, combined analysis of *BRAF* mutation and methylation markers increased the diagnostic sensitivity, ranging from 5.00 to 15.00%, and the diagnostic accuracy, ranging from 4.44 to 9.44%, with the excellent specificity, PPV and NPV (Table 4).

**Table 4:** **Diagnostic values of combined detection of *BRAF *** **mutation and various DNA methylation markers on FNAB specimens for PTC detection [percent (PTCs with *BRAF *** **mutation or methylation-positive/total PTCs, sensitivity; benign nodules with *BRAF *** **mutation or methylation-negative/total benign nodules, specificity; PTCs with *BRAF *** **mutation or methylation-positive/total positive cases, PPV; benign nodules with *BRAF *** **mutation or methylation-negative/total negative nodules, NPV)]**

	**Cut-off values**	**Training group (n =79)**				
		**Sensitivity (%)**	**Specificity (%)**	**PPV (%)**	**NPV (%)**	**Accuracy (%)**
*BRAF*^*V600E*^	N/A	71.43 (30/42)	100 (37/37)	100 (30/30)	75.51 (37/49)	71.43
*CALCA + BRAF*^*V600E*^	8.022	71.43 (30/42)	100 (37/37)	100 (30/30)	75.51 (37/49)	71.43
*DAPK1 + BRAF*^*V600E*^	0.64	78.57 (33/42)	100 (37/37)	100 (33/33)	80.43 (37/46)	78.57
*TIMP3 + BRAF*^*V600E*^	6.46	73.81 (31/42)	100 (37/37)	100 (31/31)	77.08 (37/48)	73.81
*RAR-beta + BRAF*^*V600E*^	11.21	73.81 (31/42)	100 (37/37)	100 (31/31)	77.08 (37/48)	73.81
*RASSF1A + BRAF*^*V600E*^	0.003	76.19 (32/42)	100 (37/37)	100 (32/32)	78.72 (37/47)	76.19
All five genes *+ BRAF*^*V600E*^	N/A	85.71 (36/42)	100 (37/37)	100 (36/36)	86.05 (37/43)	85.71
	**Cut-off values**	**Test group (n =38)**				
		**Sensitivity (%)**	**Specificity (%)**	**PPV (%)**	**NPV (%)**	**Accuracy (%)**
*BRAF*^*V600E*^	N/A	70.00 (14/20)	100 (18/18)	100 (14/14)	75.00 (18/24)	70.00
*CALCA + BRAF*^*V600E*^	8.022	75.00 (15/20)	100 (18/18)	100 (15/15)	78.26 (18/23)	75.00
*DAPK1 + BRAF*^*V600E*^	0.64	80.00 (16/20)	94.44 (17/18)	94.12 (16/17)	80.95 (17/21)	74.44
*TIMP3 + BRAF*^*V600E*^	6.46	75.00 (15/20)	100 (18/18)	100 (15/15)	78.26 (18/23)	75.00
*RAR-beta + BRAF*^*V600E*^	11.21	75.00 (15/20)	100 (18/18)	100 (15/15)	78.26 (18/23)	75.00
*RASSF1A + BRAF*^*V600E*^	0.003	75.00 (15/20)	100 (18/18)	100 (15/15)	78.26 (18/23)	75.00
All five genes *+ BRAF*^*V600E*^	N/A	85.00 (17/20)	94.44 (17/18)	94.44 (17/18)	85.00 (17/20)	79.44

## Discussion

Thyroid nodules are among the most common endocrine complaints in the world, which sometimes represents a significant diagnostic challenge in differentiating malignant from benign lesions. Currently, morphological diagnosis methods, such as immunohistochemistry and cytology, play an important role in differentiating malignant from benign nodules. For instance, depending on the histomorphologic features of routine hematoxylin and eosin (H&E), cytology of FNABs often provides a definitive diagnosis and sound therapeutic guidance. However, a substantial number of cases cannot be diagnosed by this technique and many cases generate indeterminate results [9]. Nowadays, the role of molecular markers in cancer diagnosis and treatment has been established, including thyroid cancer [25,26]. For example, galectin-3, fibronectin-1, CK-19, CK-903, CITED1, Ret oncoprotein, TG, Ki67, anti-MAP kinase, p16, and the mesothelial cell surface protein HBME-1 have been used as preoperative diagnostic markers [27-32]. Unfortunately, none of them are specific for thyroid malignancies.

In recent years, testing for the *BRAF*^*V600E*^ mutation has been extensively used to improve the diagnostic accuracy of thyroid malignancy in nodules, because of the high specificity of this mutation for PTC [12-14,19,25,26,33]. *BRAF*^*V600E*^ mutation, which is the most common oncogenic genetic event found in thyroid cancer, particularly in PTC, results in constitutive and oncogenic activation of BRAF kinase in the MAPK signaling pathway [10]. Through activating MAPK pathway, the *BRAF* mutation plays a fundamental role in the tumorigenesis of PTC and predicts its poor clinical outcomes [10,11]. In this study, we tested the role of *BRAF* mutation in the diagnosis of malignant thyroid nodules. The data showed that the prevalence of *BRAF* mutation was more than 70%, and was highly specific for PTCs, as supported by our data that all *BRAF*^*V600E*^-positive cases were histologically diagnosed as PTC after thyroidectomy. These observations suggest that molecular testing for *BRAF* mutation has an important impact on the diagnosis of thyroid malignant nodules, particularly PTC, as well as on decisions concerning the extent of surgery, particularly in the cases with indeterminate fine-needle aspiration cytology. Generally, some patients with indeterminate cytological findings undergo total thyroidectomy, whereas others choose to receive hemithyroidectomy. The patients in the latter group who prove to have thyroid cancer are usually advised to undergo completion thyroidectomy. Thus, for thyroid nodule patients, particularly the patients with indeterminate cytological findings, preoperative evaluation of a specific molecular marker, such as *BRAF* mutation, would be greatly helpful. In addition, accumulated evidences have demonstrated that the patients with *BRAF* mutation show frequent recurrence and resistance to radioactive iodine therapy, *BRAF* mutation may also be used as a prognostic factor [34,35]. *BRAF* mutation testing would thus be a very informative and useful tool in the management of thyroid nodules and cancers.

In addition to genetic factors, epigenetic events, such as aberrant gene methylation, play a critical role in thyroid tumorigenesis [15]. DNA methylation is one of major mechanisms of inactivation of tumor-related genes, particularly tumor suppressor genes, in cancer cells. The advantages of gene methylation as a molecular marker for the detection and diagnosis cancer in biopsy specimens and non-invasive body fluids, such as serum, has led to many studies of application in human cancers, including thyroid cancer [18]. Similarly, we hypothesize that there is potential value of gene methylation testing on FNABs as diagnostic markers to distinguish malignant from benign nodules. There are several reasons to support our hypothesis. First, DNA methylation commonly occurs in thyroid cancer [15]. Second, in generate, the amount of DNA from cancer cells in FNABs is much greater than in serum samples. Third, DNA, unlike mRNA, is a stable molecule, offering the promise of greater test stability. Fourth, DNA methylation can be quantitatively analyzed by Q-MSP assay used in this study, which has become a well-established and widely available technique [18,20,22,24]. In this study, we have confirmed the measurability of FNAB methylation markers and preliminarily defined the specificity and sensitivity of a panel of methylation markers for evaluating thyroid nodules. Using these five genes, *CALCA*, *RAR-beta*, *DAPK1*, *TIMP3,* and *RASSF1A* for differential diagnosis of thyroid nodules with appropriate cut-off values, we were able to identify positive gene methylation in 33.33-88.10% of malignant nodules, but the diagnostic specificities were all low, ranging from 75.68 to 83.78%. Although *DAPK1* and *RASSF1A* genes exhibited significantly different levels of DNA methylation between malignant and benign nodules, their diagnostic accuracy was still poor. These findings suggest that quantitative detection of these methylation markers on FNABs has serious limitations in the diagnosis of thyroid cancer.

Notably, the analysis of *BRAF* mutation confirmed the previously reported inverse association with *RASSF1A* methylation [22,24,36,37]. In addition, our data showed a positive correlation between *DAPK1* methylation and *BRAF* mutation. To our knowledge this is the first evidence indicating a relationship between these two molecular alterations in thyroid cancer. Given that both of *BRAF* mutation and gene methylation play a key role in thyroid carcinogenesis and there is certain relationship between them, we presume that combined detection of these two molecular events on FNABs may improve the diagnostic accuracy of thyroid nodules. To this end, we performed *BRAF* mutation and DNA methylation assays in training and test groups, respectively, and demonstrated that, as compared with *BRAF* mutation and DNA methylation testing each alone, combined analysis of these two molecular events increased the diagnostic sensitivity and accuracy, with excellent specificity (94.44-100%).

## Conclusion

In summary, combined detection of *BRAF* mutation and methylation markers on FNABs may be a useful strategy in evaluation of thyroid nodules with indeterminate cytological findings. Importantly, such a combined analysis will make preoperative risk and prognostic evaluation of malignant thyroid neoplasm more accurate and effective.

## Competing interests

The authors declare that they have no competing interests.

## Authors’ contributions

PH and BS conceived and designed the experiments. BZ, SL and ZZ performed the experiments. JW, YQ, KW and QY collected the samples and analyzed the data. BS and PH contributed reagents/materials/analysis tools. PH wrote the paper. All authors are in agreement with the content of the manuscript and this submission. All authors read and approved the final manuscript.
